# Supplementary material for: Unveiling structural effects on the DC conductivity of warm dense matter via terahertz spectroscopy and ultrafast electron diffraction
Source: Nat Commun. 2025 Nov 26;16:10541. doi: 10.1038/s41467-025-65559-5 (PMC12658010; doi:10.1038/s41467-025-65559-5)
Supplement: Supplementary file 1 — Supplementary Information [file 41467_2025_65559_MOESM1_ESM.pdf]

Supplementary Information

Unveiling Structural Effects on the DC Conductivity Warm Dense Matter via Terahertz Spectroscopy and Electron Diffraction

Benjamin K. Ofori-Okai[1,2*], Adrien Descamps[1,3,4], Edna R. Toro[1,5], Megan Ikeya[1], Stephanie B. Hansen[6], Mianzhen Mo[1], Andrew D. Baczewski[7], Danielle Brown[1,8], Luke B. Fletcher[1], Emma E. McBride[1,4], Xiaozhe Shen[1], Anthea Weinmann[1,9], Jie Yang[1], Jochen Schein[9], Zhijiang Chen[1], Xijie Wang[1,10,11], Siegfried H. Glenzer[1]

[1]SLAC National Accelerator Laboratory, Menlo Park, CA 94025, USA

[2]PULSE Institute, SLAC National Accelerator Laboratory, Menlo Park, CA 94025, USA

[3]Aeronautics and Astronautics Department, Stanford University, Stanford, CA 94305, USA

[4]School of Mathematics and Physics, Queen's University Belfast, Belfast BT7 1NN, UK

[5]Mechanical Engineering Department, Stanford University, Stanford, CA 94305, USA

[6]Pulsed Power Sciences Center, Sandia National Laboratories, Albuquerque, NM 87123, USA

[7]Center for Computing Research, Sandia National Laboratories, Albuquerque, NM 87123, USA

[8]Physics Department, Stanford University, Stanford, CA 94305, USA

[9]Universität der Bundeswehr München, 85579 Neubiberg, Germany

[10]Faculty of Physics, University of Duisburg-Essen, 47048 Duisburg, Germany

[11]Department of Physics, TU Dortmund University, 44227 Dortmund, Germany

* To whom correspondence should be addressed; Email: benofori@SLAC.stanford.edu, glenzer@SLAC.stanford.edu

Supplementary Text

Note 1. Determining $\Delta t = 0$ between optical drive and THz probe

To determine pump-THz temporal overlap ($\Delta t = 0$), optical-pump THz probe measurements were made on Si samples below the damage threshold. The setup is shown in Fig. S1(a). The optical drive pulse was attenuated and overlapped with the THz pulse at the sample position. The time-delay was scanned and THz waveforms were collected for each Δt. Example traces are shown in Fig. S1(b). Figure S1(c) shows a complete dataset collected for analysis. The peak amplitude of the THz field was extracted as a function of Δt, $S(\Delta t)$, and this was fit to a modified error function,

$$S(\Delta t) = \frac{A}{2} \int_{\Delta t}^{\infty} \exp\left(- \left[\frac{\tau - \Delta t_0}{w} \right]^2 \right) d\tau + C. \tag{S1}$$

An example of the extracted $S(\Delta t)$ and the corresponding fit are shown in Fig. S1(d). From the fit, we extracted Δt_0, with $w = 0.3$ ps defining the error.

Note 2. Comparison between real and imaginary parts of the conductivity

Figure S2 shows the ratio of the real and imaginary parts of the conductivity extracted from the THz measurements. The contribution from the imaginary part of the conductivity to the absolute value is given by equation (S2):

$$|\sigma| = \sigma_r \sqrt{1 + \left(\frac{\sigma_i}{\sigma_r} \right)^2}. \tag{S2}$$

For the largest case of $\sigma_i/\sigma_r = 0.01$, the total difference between the real and absolute values is <1%, which is considerably smaller than our error. In the context of the Drude model for the conductivity, the real and imaginary parts are given by:

$$\tilde{\sigma}(\omega) = \frac{\sigma_0}{1 - i\omega/\nu_e} = \frac{\sigma_0}{1 + (\omega/\nu_e)^2} + i \frac{\sigma_0 \omega/\nu_e}{1 + (\omega/\nu_e)^2}. \tag{S3}$$

From this expression and estimates of the scattering rate, we expect $\sigma_i/\sigma_r = \omega/\nu_e \sim 0.01$, supporting the Drude-like behavior of WD-Al for the conditions investigated.

Note 3. Determining sample thickness

To determine the thickness of the Al layer for the samples used in the THz measurements, different sections of the sample card were measured using a Helios focused ion beam combined with a scanning electron microscope (FIB-SEM). The film was coated with a sacrificial layer of ~600 nm of platinum or amorphous carbon and then milled using a Helios FIB to yield a cross section of the sample. This cross section was imaged using the SEM in immersion mode at 1 kV voltage and a current of 43 pA, which yielded regions showing the deposited layer, the Al, and the silicon wafer and silicon nitride layers. These images were analyzed to find the edges between the carbon and the Al, and the Al and Si/Si_3N_4 layer. An example image in shown in Fig. S3. The number of pixels between the edges was measured and converted into a distance using the scale. Various positions on the sample card were measured to obtain statistics. The results of the different runs are tabulated in Table S1.

Run number	Thickness (nm)
**1**	27.1
**2**	30.0
**3**	30.6
**4**	32.4
**5**	33
**6**	37
**7**	41.1
**Average**	33.0
**Standard deviation**	4.7

Table S1. Measurements of the film thickness from FIB-SEM.

Note 4. Determining sample expansion from Frequency Domain Interferometry

The evolution of the sample thickness was measured using Frequency Domain Interferometry[1,2]. Figure S4 shows a schematic of the setup. The experiments were performed in a vacuum chamber

to ensure that high intensity pulses could be directed onto the sample. The drive pulses were λ = 400 nm ultrafast laser pulses with a duration of ~ 50 fs generated by frequency doubling the fundamental λ = 800 nm pulses produced by a Ti:Sapphire laser. These pulses were at normal incidence to the foil. The doubling was performed using a 200 μm thick β-barium borate (BBO) crystal. A pair of time-delayed and spectrally chirped (duration ~ 15 ps) λ = 800 nm pulses, the reference and the probe, derived from the same Ti:Sapphire laser system were directed at the foil at a 45° angle and were polarized to 45 (equal mixtures of S- and P- polarization). The time-delay between the probe pulses was ~ 12 ps, and the pump pulse arrived between the reference and probe pulses.

The reflected 800 nm pulses were then directed through a 10° splitting Wollaston prism set to transmit S- and P-polarized beams and finally into an imaging spectrometer. This resulted in a pair of interferogram images resulting from the temporal delay between the reference and the probe. Changes in the interferogram were used to extract the phase of the S- and P- components probe, ϕ_s and ϕ_p respectively, which arise due to changes in the target after irradiation by the drive. As the target expands, there is an induced doppler shift in the probe, $\delta\phi$, which can be calculated according to

$$\delta\phi = 2\phi_s - \phi_p, \tag{S4}$$

and the doppler shift can be related to the expansion of the sample, Δx,

$$\delta\phi = \frac{4\pi\cos(\theta)\Delta x}{\lambda}. \tag{S5}$$

Figure S4(b) shows the extracted expansion at different times for different energy densities. The data were fit to lines which yield an expansion velocity for different energy densities. These expansion velocities were parameterized according to the function,

$$v \approx 0.5847\ln(\rho_E) + 0.4123. \tag{S6}$$

The data and the corresponding fit are shown in Fig S4(c).

Note 5. Background correction procedure for UED data

Figures S5 and S6 show example false color images collected during the MeV-UED experiments as well as the procedure for background subtraction. For each time delay, the diffraction was unwrapped using the OpenCV Python package to convert the 2D map, $I(x,y)$ into an $I(Q,\phi)$ map. This map was then averaged along ϕ to produce a radially integrated profile, $I(Q)$. Figure S4(a) shows example plots, offset for clarity, of unpumped films for one fluence.

To determine the background, the intensity of the pattern for $1.5 < Q < 2$, $3.5 < Q < 4$, and $4.5 < Q < 5.75$ were isolated and fit to the following background function,

$$B(Q) = A\exp(-kQ) + C, \tag{S7}$$

to determine a set of coefficients A, k, and C. The effectiveness of this background subtraction is illustrated in Fig. S5(b). For the corresponding pumped sample, this background function was subtracted from the radially integrated plot. Example uncorrected and corrected $I(Q)$ are shown in Fig. S5(c) and (d), respectively. Figure S6 shows the background corrected radial lineouts for all data used in this analysis.

Note 6. Determining the width of the liquid diffraction based on previous measurements

To determine expected scattering pattern for liquid Al, it is necessary to convert the static structure factor to the measured scattering intensity. In mega electron volt ultrafast electron diffraction (MeV-UED), the measured intensity, $I(Q)$, is related to the static structure factor, $S(Q)$, the electron form factor, $f_e(Q)$, and the Lorentz factor, γ, and the instrument response function, $R(Q)$, which accounts for a number of effects including the spreading of the electron bunch due to space charge and the imaging of the phosphor screen onto the EMCCD. The measured signal can be determined according to

$$I(Q) = [S(Q)(\gamma f_e(Q))^2] * [R(Q)], \tag{S8}$$

with $f_e(Q)$ computed as a sum of Gaussians using coefficients a_n and b_n taken from calculations[3]. For Al^{3+},

$$f_e(Q) = \sum_{n=1}^{5} a_n \exp\left(-b_n \left[\frac{Q}{4\pi}\right]^2\right), \tag{S9}$$

and $S(Q)$ taken from neutron scattering measurements of liquid Al near the melting temperature[4,5]. Figure S7(a) shows $f_e(Q)$ and $S(Q)$ used, where $\gamma f_e(Q)$ has been multiplied by 0.1 to be comparable to $S(Q)$.

The resolution function was determined from measurements of solid single crystal Al measured on using the UED instrument. A pattern from the single crystal is shown in Fig. S7(B) and the result of azimuthally integrating the pattern is shown in Fig. S7(c). The red curve in Fig. S7(c) shows a Gaussian fit to the lowest order peak, in this case the (200) reflection, with a corresponding FWHM of 0.28 $Å^{-1}$.

Figure S7(d) shows the result of applying equation S7 using the data presented, along with a fit to the lowest order peak in the calculated $I(Q)$. The dashed black line is $R(Q)$, multiplied by 0.5, shows the effect of broadening that would be expected for the liquid. The fit to the liquid signal has a FWHM of 0.67 $Å^{-1}$, and this value is used in the manuscript (see main Fig. 2).

Note 7. Evaluating error in temperature estimates

We determined the error in our temperatures by error propagation through the Two-Temperature model calculations. The primary source of error was attributed to the input energy density, ρ_E, which is related to the laser fluence, F, the exposed area, A, the initial sample thickness, d_0, and the mass density, ρ_M, according to:

$$\rho_E = \frac{FA}{d_0 \rho_M}. \tag{S10}$$

Here, we assume that A and ρ_M are fixed parameters and consider the uncertainties in F and d_0, δF and δd_0, respectively. Assuming that δF and δd_0 are uncorrelated, we determine the variance in the energy density as:

$$\delta\rho_E = \rho_E\sqrt{\left(\frac{\delta F}{F}\right)^2 + \left(\frac{\delta d_0}{d_0}\right)^2}. \tag{S11}$$

To determine the value for δF, we considered variation in the shot-to-shot laser intensity and the spatial variation in the beam. The RMS error in the shot-to-shot laser intensity was ~1%, determined by measuring the statistics of the laser energy at the sample position over 250 shots. To determine error caused by spatial heterogeneity, a representative measurement of the laser spot at the sample plane was recorded, as shown in Fig. S8(a), and the intensity variation over a 600 × 600 μm^2 region was determined. An intensity histogram was measured, shown in Fig. S8(b), and the RMS intensity fluctuations were determined to be ~20%, dominating the contribution of the error in δF. For δd_0, we determined a 14% error, leading to an overall error of $\delta\rho_E/\rho_E$ of ~24%. Finally, for each energy density ρ_E, the errors in the electron and ion temperatures, ΔT_e and ΔT_i, were determined by extracting outputs from the TTM simulations for $\rho_E \pm \delta\rho_E$.

Note 8. DFT-AA conductivity calculations

Figure S9 shows examples of various components of the Ziman integral that are used to compute the electrical conductivities shown in Fig. 6 of the main text. Figure S9(a) shows the cross sections, multiplied by the Ziman Q^3 weighting factor, for aluminum at 2.7 g/cm^3 at various electron temperatures. Here we assume a Slater exchange potential (a 3/2 factor on the simple Kohn-Sham LDA exchange potential). As seen, increasing electron temperatures tend to broaden the scattering peaks. Integrating the product of these Q-weighted cross sections with the static ion structure factor $S_{ii}(Q)$ gives an electron-ion scattering frequency, ν_{ei}, that is inversely proportional to the DC conductivity.

Figure S9(b) shows calculations of the structure factors for the liquid (T_i = 1 eV) and solid ($T_i \leq$ 0.1 eV) phases for aluminum at 2.7 g/cm^3 and T_e = 1 eV. For liquids, we use the self-consistent ion structure factor from the quantum Orenstein-Zernike (QOZ) equations[6], excluding elastic

scattering from incipient crystalline structure[7] by restricting $S_{ii}(Q)$ to be smaller than unity. For solids, we follow Potekhin *et al.*[8] to approximate the part of the static ion structure factor that contributes to inelastic scattering in solids, excluding contributions from the elastic Bragg peaks. While changing electron temperatures have a modest impact on the Ziman integral, changing ion temperatures can have a profound effect on ν_{ei} and the DC conductivity.

Note 9. Comparison of effect of constant pressure vs constant volume heat capacities on TTM

Figure S10 shows the results of TTM calculations using the constant volume and constant pressure heat capacities. The constant pressure heat capacity is taken from the NIST database and is parametrized by the ion temperature using the Shomate equation:

$$C_p = A + BT_i + CT_i^2 + DT_i^3 + ET_i^{-2}. \quad \text{(S12)}$$

The constants *A, B, C, D,* and *E* are specified for the solid and liquid phase. The constant volume heat capacity is investigated in two cases: 1) the value from the Dulong-Petit limit, C_v^0, and 2) the temperature dependent C_v using the Debye model. In this case,

$$C_v(T_i) = 3C_v^0 \left(\frac{T}{T_D}\right)^3 \int_0^{T_D/T} \frac{x^4 e^x}{(e^x - 1)^2} dx. \quad \text{(S13)}$$

Here T_D = 428 K is the Debye temperature of Al. As expected, the higher constant pressure heat capacity results in a lower final temperature of the system. The absolute difference is largest for the highest energy density simulated and corresponds to an ~5% difference.

Note 10. Consolidating conductivity measurements

Figure S11 shows a plot of the conductivity compared to the electron and ion temperatures. The labels for the points show the conductivity scaled to account for the hydrodynamic expansion described in the next section.

Note 11. Scaling conductivity to solid density

Figure S12 shows a reproduction of Fig. 6 from the main manuscript, where the data from the THz measurements has been scaled according to the linearity of the density, as well as the unscaled values determined directly from the THz measurements. The scaling factor was determined using the FDI data and the film expansion was assumed to be only in the lateral dimension. For a given

thickness d after expansion, the final mass density, ρ, is related to the initial mass density, ρ_0, and the initial sample thickness, d_0, according to

$$\rho = \rho_0 \frac{d_0}{d}. \tag{S14}$$

As the conductivity is expected to be linearly proportional to the carrier density, n_e, and the carrier density is proportional to the mass density, the scaled conductivity, σ_s, can be derived from the measured density σ_M, according to

$$\sigma_s = \sigma_M \frac{d}{d_0}. \tag{S15}$$

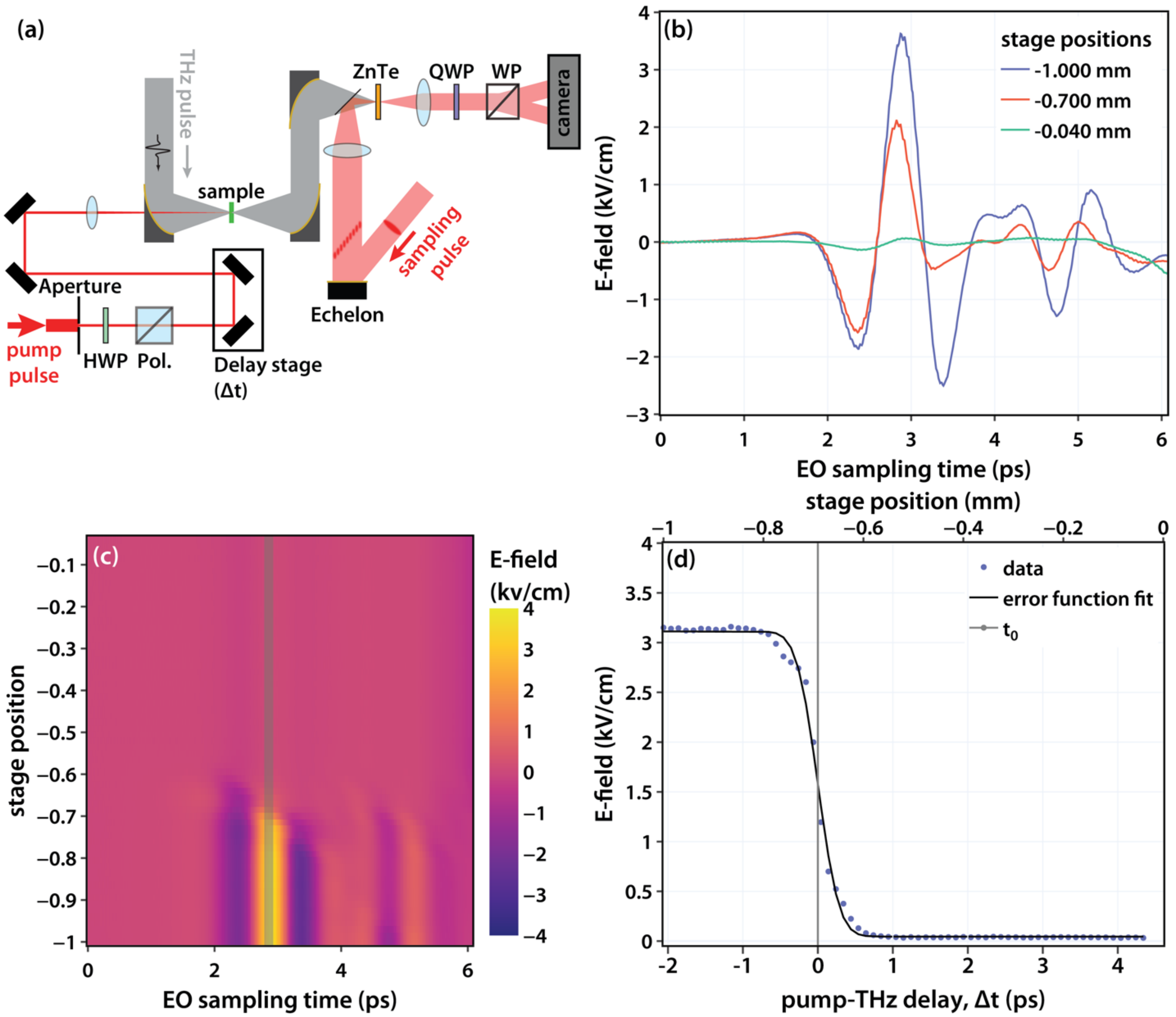

Fig. S1. Setup for THz measurements and determination of Δt = 0. In **a** is a schematic of the setup used for determining $\Delta t = 0$. The THz, pump, and sampling pulses are all derived from the same laser source. Plots of THz time-domain waveforms in **b** measured for different delay stage positions, corresponding to different Δt. Image in **c** is a 2D map showing the variation in the THz waveforms for different stage delays. The transparent region around 3 ps in the EO sampling time was averaged over to produce a lineout. The plot in **d** is a lineout and fit to the data according to the equation S1.

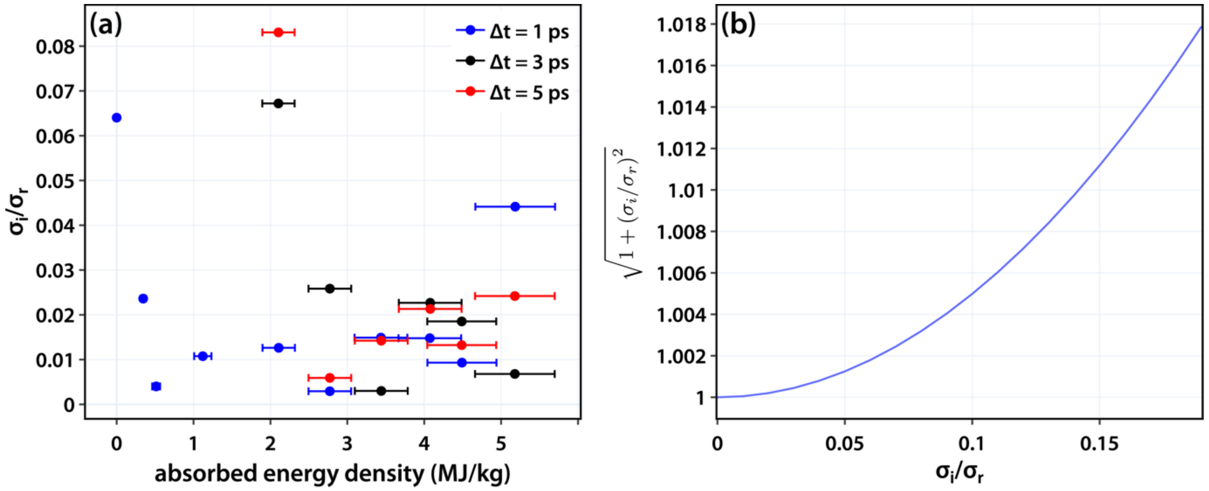

Fig. S2. Comparison between real and imaginary conductivity. In **a** is a plot of the ratio of the imaginary to the real part of the conductivity extracted from the THz measurements. Each single point is derived by averaging over the same spectral range as shown in main manuscript Fig. 4. The relative importance of the imaginary part of the conductivity compared to the real part is shown in **b**. For the $\sigma_i/\sigma_r \sim 1$ measured, the relative contribution is < 0.01.

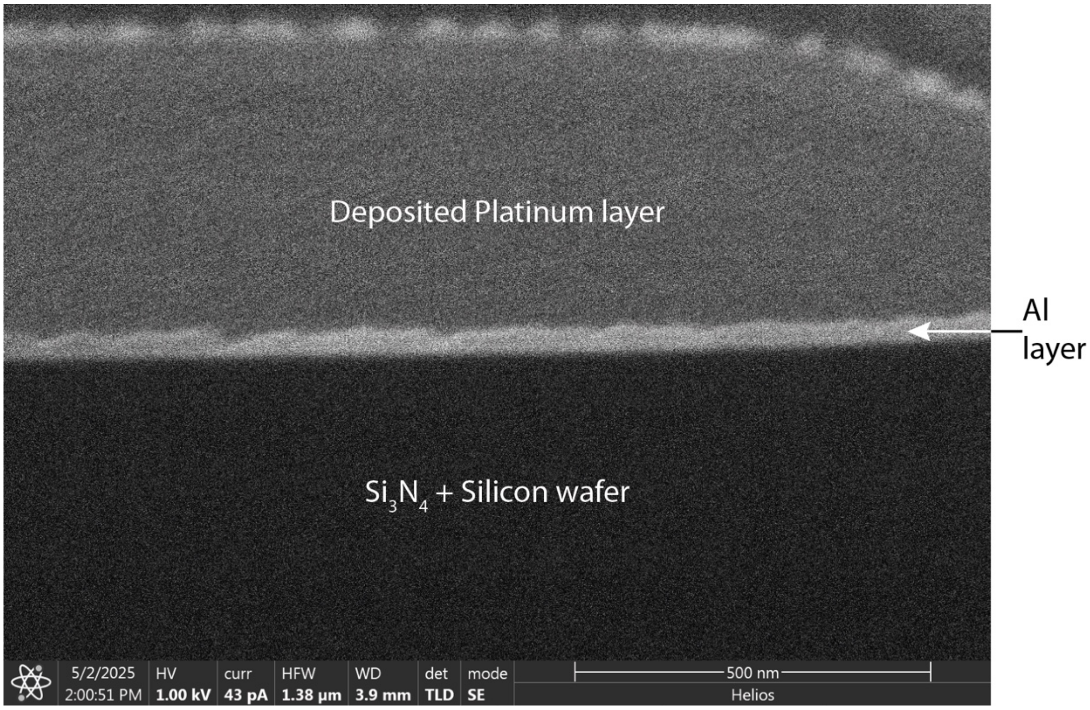

Fig. S3. Representative SEM image of cross section of film coated with platinum.

SEM image showing the deposited platinum layer (medium gray) on top of the Al layer (light gray) and the Si/Si3N4 layer (dark gray).

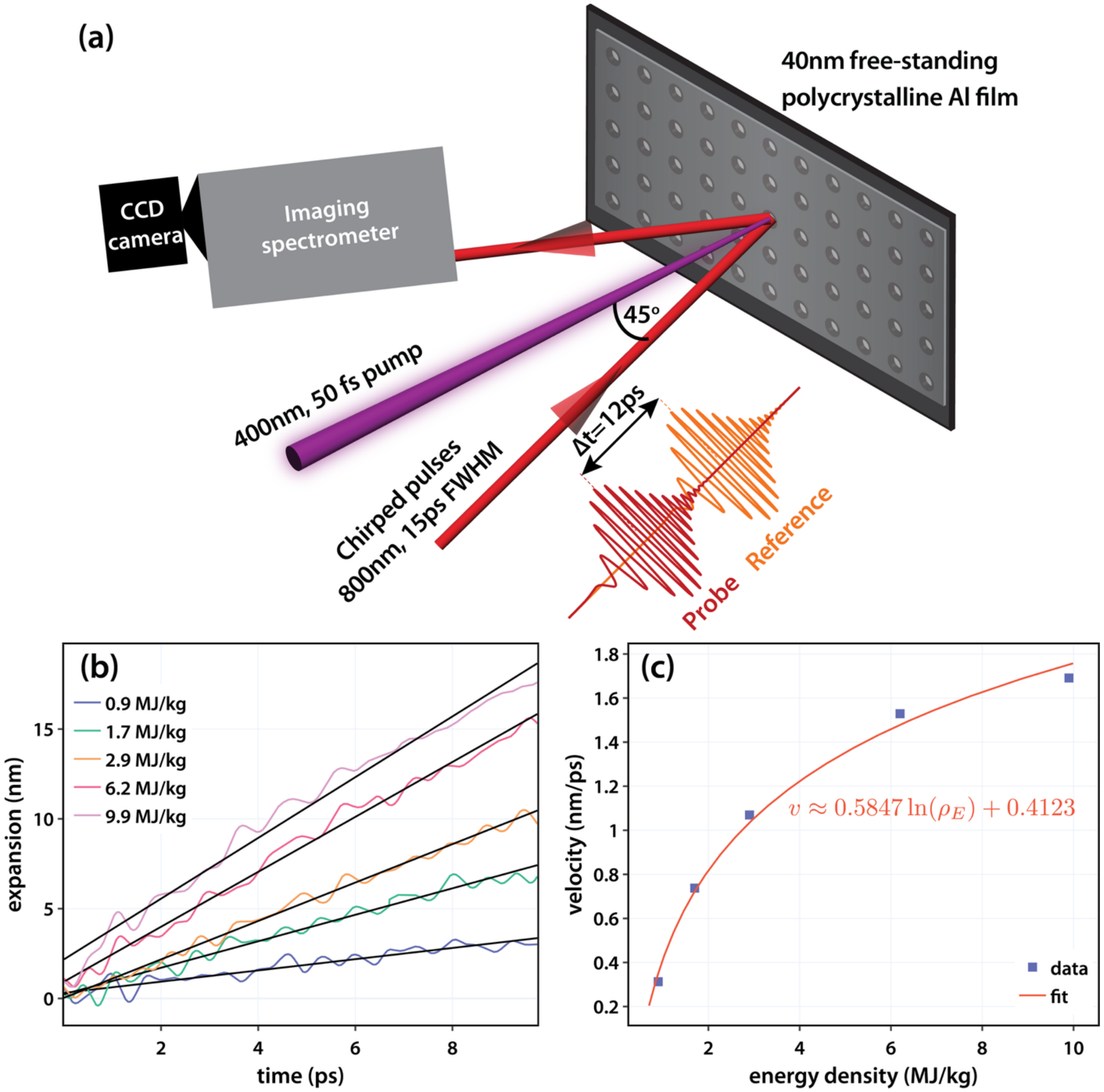

Fig. S4. Frequency domain interferometry measurements on Al. Schematic illustration in **a** shows the setup for FDI measurements. **b** shows the film expansion derived from FDI measurements with linear fits for different energy densities. The expansion here is for a single side of the film. In **c** are the expansion velocities derived from **b** for different energy densities, with a fit to a logarithm function. The R^2 for the fit was 0.9924.

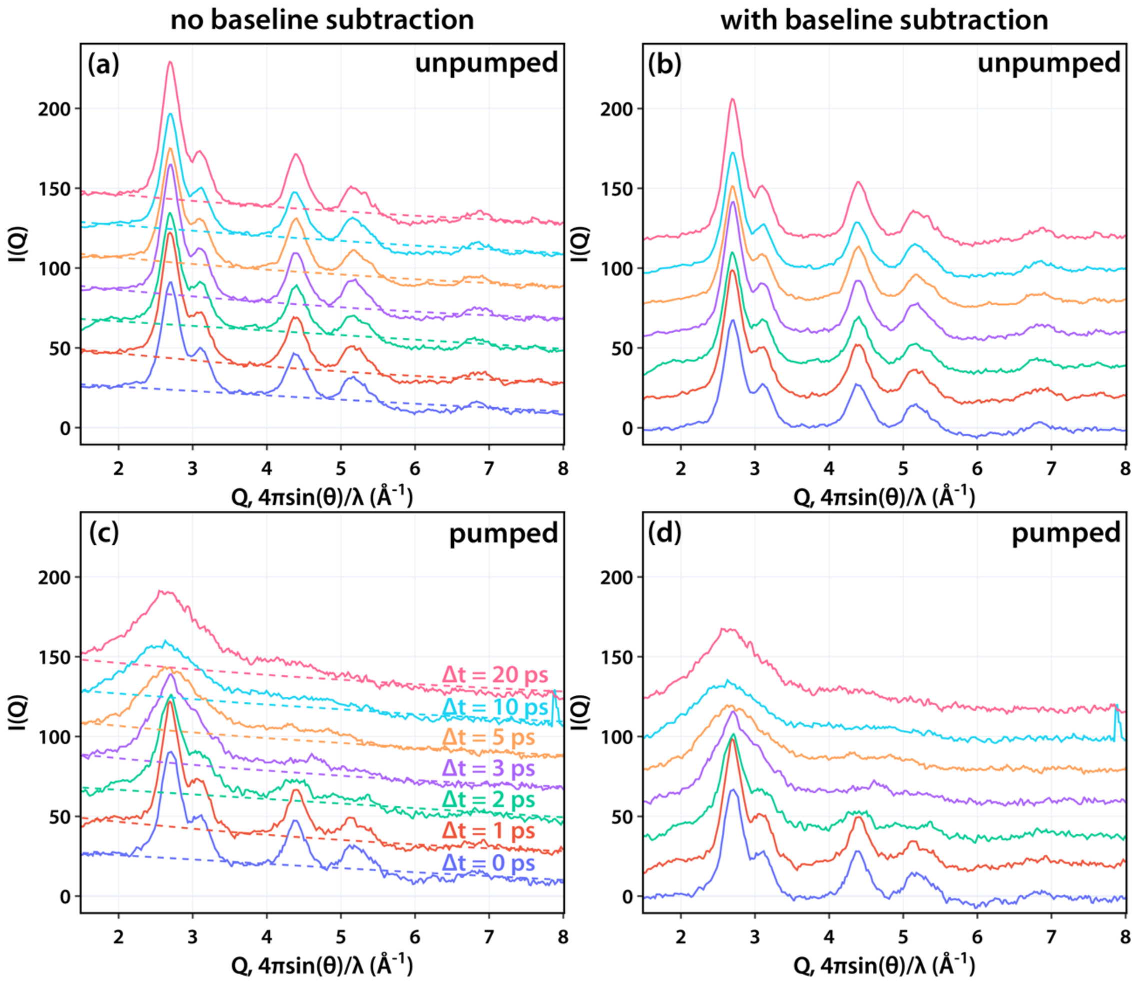

Fig. S5. Illustration of background correction procedure for UED data. Azimuthally integrated patterns in **a** for unheated films at different time delays, vertically offset for clarity. The dashed lines indicate the background resulting from fitting the signal away from the diffraction peaks. In **b** are the results of background subtraction of the azimuthally integrated patterns in **a**. In **c** are azimuthally integrated patterns for pumped films at different time delays, vertically offset for clarity. The dashed lines are the backgrounds extracted from the corresponding measurements on unpumped films. Curves in **d** are background-subtracted azimuthally integrated patterns.

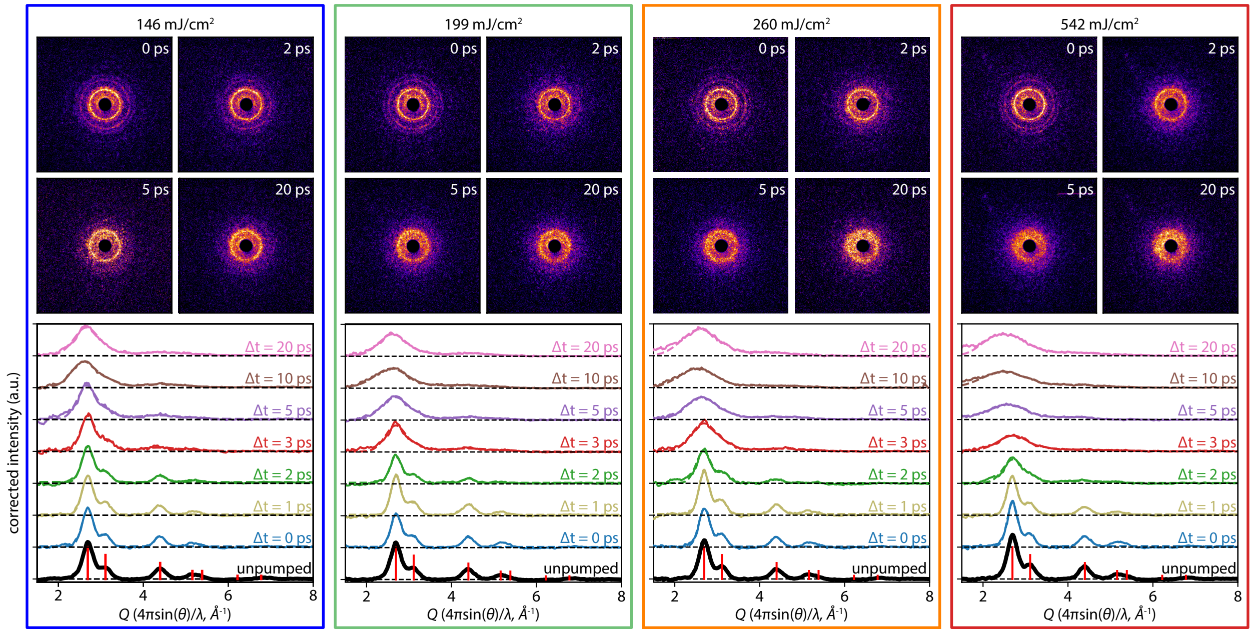

Fig. S6. Variation in time-resolved electron diffraction with laser fluence. False color diffraction patterns for select time delays, and background-subtracted azimuthally integrated patterns at all time delays for MeV-UED data.

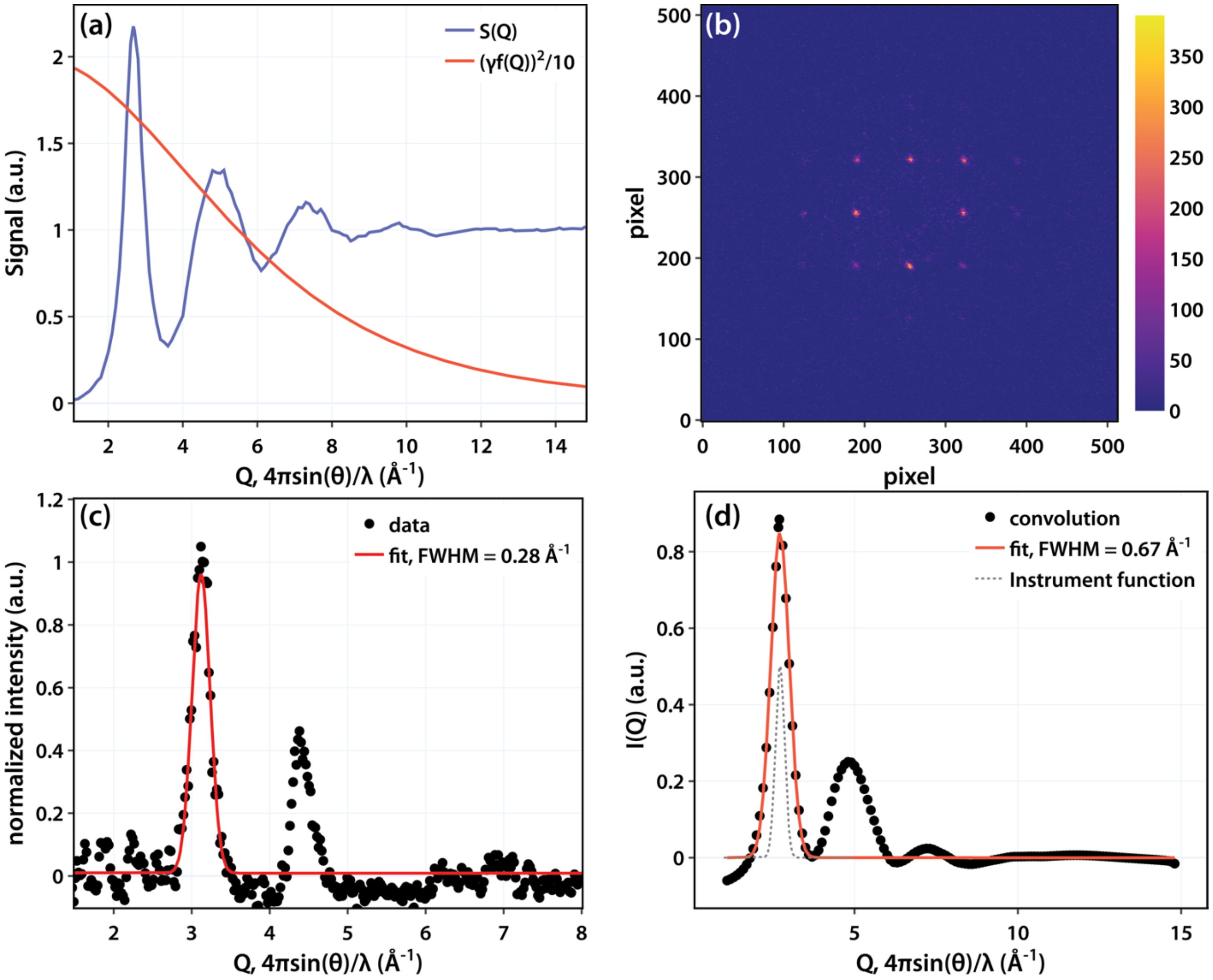

Fig. S7. Determination of electron diffraction for liquid Al using neutron scattering data. Plots in **a** of the static structure factor, $S(Q)$, for liquid as determined by neutron scattering measurements and $\gamma f(Q)^2$ determined from literature. Image in **b** is a false color diffraction pattern of single crystal aluminum. Data in **c** shows the result of azimuthal integration of **b**. The dots are the data and the red line is a Gaussian fit to the (200) diffraction peak, which defines the resolution function for the UED instrument. The reconstructed $I(Q)$ using equation S7 is shown in **d**, along with a fit to the lowest order feature (red line) and the instrument (dashed line). The width to the lowest order feature extracted from the fit is used to determine when the sample has transitioned to the liquid state.

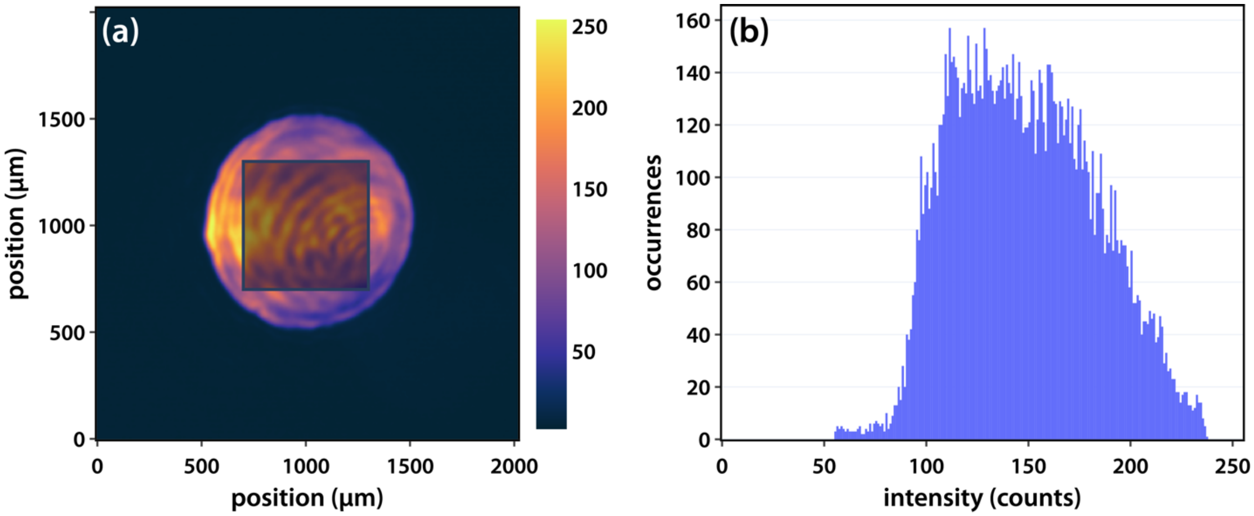

Fig. S8. Details of drive beam and contributions to error. Image in **a** of the laser spot at the sample for the THz measurements. The translucent square is the 600 x 600 μm spot probed by the THz pulse. A histogram of the intensity values within the region probed by the THz pulse is shown in **b**.

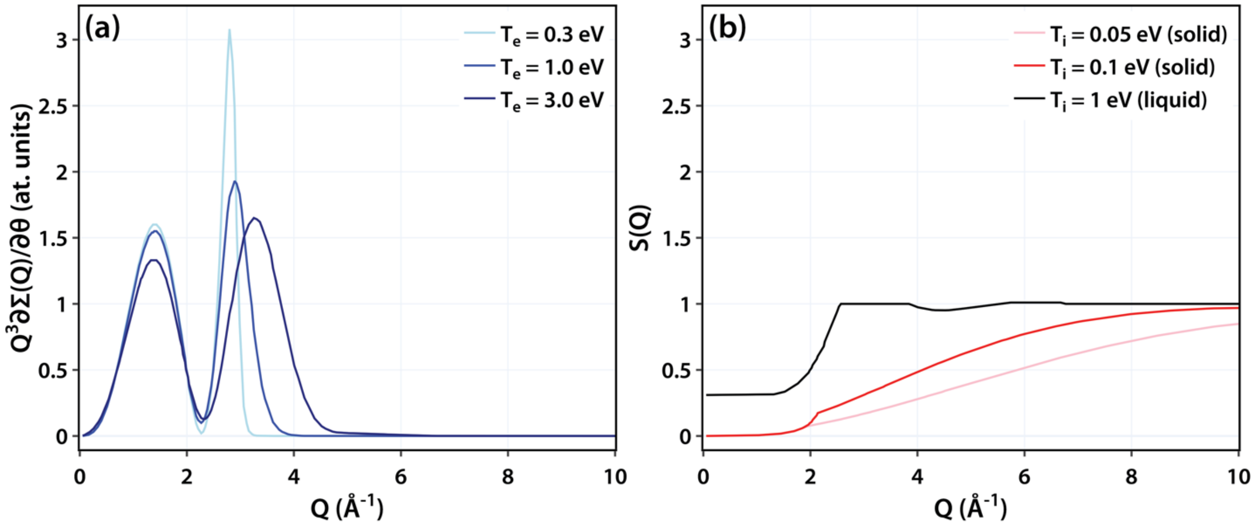

Fig. S9: Components of the *Q*-dependent Ziman integral, used to generate the DFT-AA conductivities. Plots in **a** show *Q*-weighted, energy-integrated cross sections following [9], which depend primarily on the electron temperature. The static ion structure factors used in the calculations are shown in **b** which depend primarily on the ion temperature. In the present equilibrium calculations, we use the $S(Q)$ prescription from Baiko and Yakovlev [7,8] for $T_i < 933$ K (the melting temperature) and the self-consistent liquid structure factor $S_{QOZ}(Q)$ following [6] for $T_i > 933$ K, excluding incipient elastic peaks by setting $S(Q) = \min[S_{QOZ}(Q),1]$.

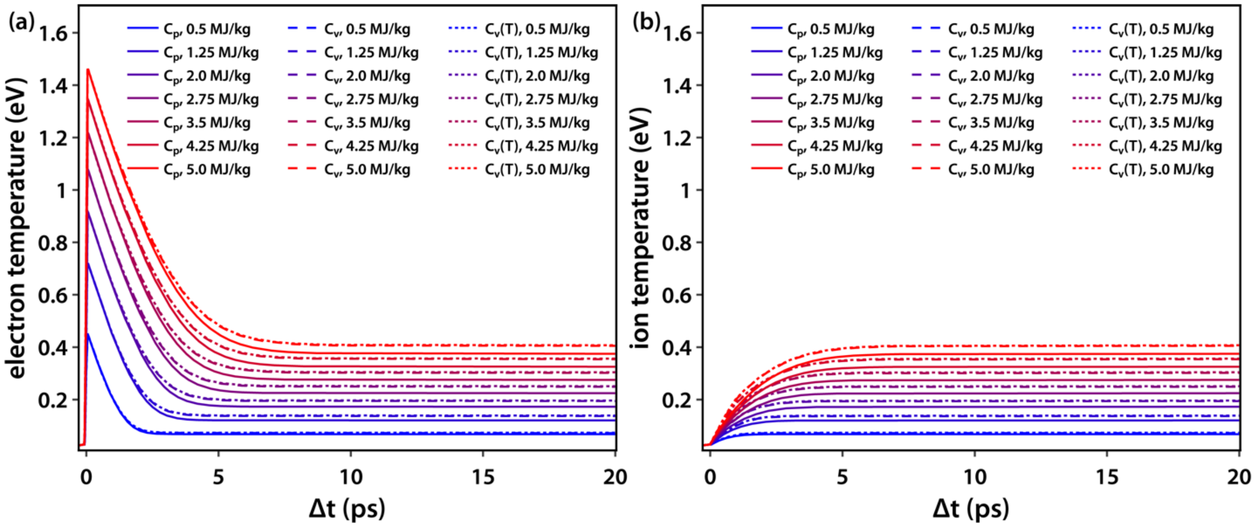

Figure S10. Comparison of constant pressure and constant volume heat capacities on TTM calculations. Results of calculations of the electron temperature are shown in **a**; corresponding plots of the ion temperature are shown in **b**. Solid lines show the results of TTM calculations using the constant pressure heat capacity, dashed lines are for the constant volume heat capacity in the Dulong-Petit limit, and dotted lines are using the $C_v(T_i)$ based on the Debye model.

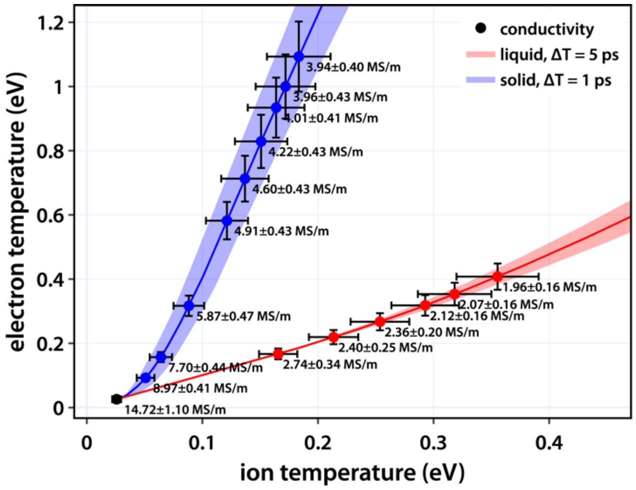

Figure S11. Consolidated TTM calculations with labeled points. The blue colored band is for the case where $\Delta t = 1$ ps, and is equivalent to using the values between the blue dotted lines shown in main Fig. 5 for the *x*-axis, and the blue solid lines shown in main Fig. 5 for the *y*-axis; the red band is the equivalent for Δt = 5ps. The dots on the plot are the T_e and T_i values where the measurements were made; the labels are the conductivities.

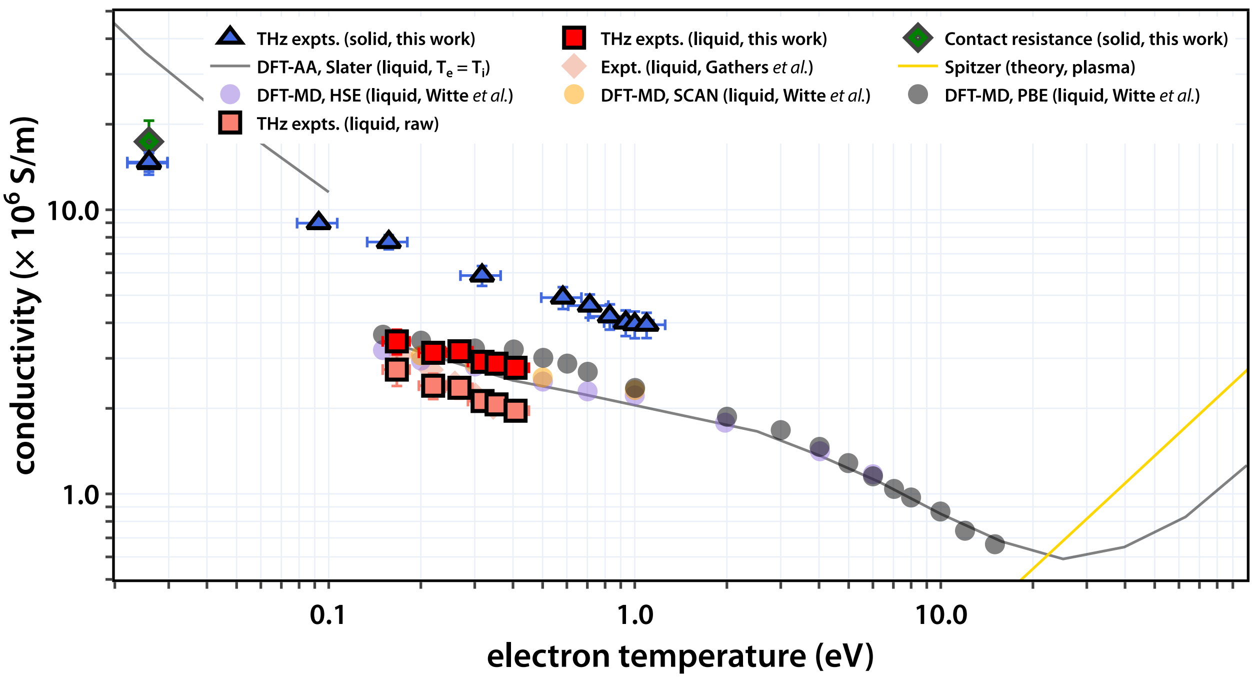

Figure S12. Comparison of conductivity determined from THz measurements with past studies. Blue triangles represent values extracted from THz measurements on Al with a solid structure and red squares are from THz measurements with a liquid structure. DFT-MD calculations by Witte *et al.* and DFT-AA calculations use a liquid structure at solid density ($\rho_0 = 2.7$ g/cm^3). For the THz measurements on liquids, the red squares represent the THz extracted conductivity scaled to 2.7/cm^3. The salmon-colored squares are the unscaled data. The Gathers' data are also scaled to solid density.

References

1. Ao, T. et al. Optical Properties in Nonequilibrium Phase Transitions. *Phys. Rev. Lett.* **96**, 055001 (2006).
2. Chen, Z. et al. Interatomic Potential in the Nonequilibrium Warm Dense Matter Regime. *Phys. Rev. Lett.* **121**, 075002 (2018).
3. Peng, L.-M., Dudarev, S. L. & Whelan, M. J. *High Energy Electron Diffraction and Microscopy*. (Oxford University Press on Demand, 2004).
4. Fessler, R. R., Kaplow, R. & Averbach, B. L. Pair Correlations in Liquid and Solid Aluminum. *Physical Review* **150**, 34–43 (1966).
5. Stallard, J. M. & Davis, C. M. Liquid-aluminum structure factor by neutron diffraction. *Phys. Rev. A* **8**, 368–376 (1973).
6. Starrett, C. E. & Saumon, D. A simple method for determining the ionic structure of warm dense matter. *High Energy Density Physics* **10**, 35–42 (2014).
7. Baiko, D. A., Kaminker, A. D., Potekhin, A. Y. & Yakovlev, D. G. Ion Structure Factors and Electron Transport in Dense Coulomb Plasmas. *Phys. Rev. Lett.* **81**, 5556–5559 (1998).
8. Potekhin, A., Baiko, D., Yakovlev, D. & Haensel, P. Transport properties of degenerate electrons in neutron star envelopes and white dwarf cores. *Astronomy and Astrophysics* **346**, 345–353 (1999).
9. Sterne, P. A., Hansen, S. B., Wilson, B. G. & Isaacs, W. A. Equation of state, occupation probabilities and conductivities in the average atom Purgatorio code. *High Energy Density Physics* **3**, 278–282 (2007).
